# Effects of marking methods and fluorescent dusts on *Aedes aegypti* survival

**DOI:** 10.1186/1756-3305-7-65

**Published:** 2014-02-12

**Authors:** Borame L Dickens, Hayley L Brant

**Affiliations:** 1 Centre for Environmental Policy, Imperial College London, South Kensington Campus, London SW7 1NA, UK; 2 Centre for Environmental Policy, Imperial College London, Silwood Park Campus, Buckhurst Road, Ascot, Berkshire SL5 7PY, UK

**Keywords:** *Aedes aegypti*, Fluorescent dust, Fluorescent paint, Survivorship

## Abstract

**Background:**

Tracking the movement of mosquitoes and understanding dispersal dynamics is essential for the control and prevention of vector-borne diseases. A variety of marking techniques have been used, including dusts and dyes.

**Methods:**

In this study, *Aedes aegypti* were marked using fluorescent dusts (‘DayGlo’: A-19 Horizon Blue & A-13-N Rocket Red; ‘Brian Clegg’: pink, blue & red), fluorescent paints (‘Brian Clegg’: blue, red & yellow) and metallic gold dust (‘Brian Clegg’). Dusting methods were those previously used in mark-release-recapture experiments, including application with a bulb duster, creation of a dust storm or shaking in a bag.

**Results:**

Results showed marking mosquitoes using a dust storm allowed relatively high survival, compared to unmarked controls (Males: χ^2^ = 3.24, df = 4, p = 0.07; Females: χ^2^ = 3.24, df = 4, p = 0.04), and high marking efficiency. Using a bulb duster showed high survival in male mosquitoes (χ^2^ = 12.59, df = 4, p < 0.000), but low survival in female mosquitoes during the first 15 days of the study (χ^2^ = 5.17, df = 4, p < 0.05). The bulb duster also had the lowest marking efficiency compared to other dry marking techniques. The bag method showed low survival in males during the first 15 days of the study (χ^2^ = 5.77, df = 4, p < 0.05). Applying paints had an overall negative impact on survival for males (χ^2^ = 5.03, df = 3, p < 0.05), but not for females (χ^2^ = 0.19, df = 3, p = 0.661). Males dusted with DayGlo Horizon Blue dust, and females dusted with DayGlo Rocket Red dust, had the most significant reduction in survivorship in comparison to the control (Males: χ^2^ = 15.70, df = 6, p < 0.000; Females: χ^2^ = 24.47, df = 6, p < 0.000). Mosquitoes marked with Brian Clegg gold dust showed mortality rates similar to controls within male mosquitoes (χ^2^ = 0.18, df = 6, p = 0.674), but significantly lower in females (χ^2^ = 16.59, df = 6, p < 0.000).

**Conclusions:**

This study showed that marking technique and colour can have a significant impact on the survival and marking coverage of a mosquito.

## Background

Dispersal is an essential component of understanding insect biology, behaviour, life history and population dynamics [1]. Understanding dispersal dynamics and flight ranges of mosquito vectors is essential for the mitigation of disease, successful implementation of protection against infection and an important step in understanding the ecology of a vector [2]. Mark-release-recapture (MRR) techniques can be used to estimate mosquito population densities, feeding behaviour, duration of gonotropic cycles and their dispersal behaviour [2]. An ideal marking technique should be cost-effective, easily applied, visible, non-toxic and should not affect the behaviour, development, longevity or reproduction of mosquitoes [1,3]. Marking techniques have the potential to affect the mortality and dispersal rates of marked individuals in a way that could bias the results arising from MRR experiments, so preliminary experiments on marking methodologies are required prior to carrying out MRR experiments [2].

A variety of methods have been used to mark mosquitoes, including dusts [4-9], dyes [10,11], paints [12,13], trace elements [14] and radioactive isotopes [15,16] (see reviews in [1-3]). One of the most common methods of marking mosquitoes externally is to apply micronized particles of dust (also known as powder or pigments), particularly fluorescent dust, to a large number of mosquitoes [1]. Dusts are cost-effective, available in a range of colours, easily applied and very detectable.

Several types of fluorescent dusts have been used to mark insects, including ‘Helecon’ and Radiant dusts, but the ‘DayGlo’ series A and AX are now the most commonly used as no adhesive is needed for mosquitoes to retain marks [2]. Dusts can be applied using a syringe [7] or bulb duster [17], putting them in a bag containing dust and shaking them gently [18], or by creating a dust storm within a cage [19]. Many shaking methods can cause high mortality by applying too much dust to mosquitoes. This can increase mortality, decrease mobility and affect the sensory organs [1], giving biased results in MRR studies. There is also the constraint of dusts not persisting long enough for long-term studies and transference of dusts to unmarked individuals [1,20].

Paints can be applied individually [12] or to large groups of individuals [11,13]. Applying paints individually can be time-consuming, but mass marking by spraying paints can be easy and quick. Usually paints are diluted with acetone or alcohol before being sprayed from hand atomizers or spray guns [1]. Myles and Grace [21] experimented with spray paints as an adhesive for borate dusts on termites, claiming they were non-toxic. Fluorescent paints adhere to body parts, can achieve 100% coverage and are readily identifiable under UV light [22]. Marking with paints is usually believed to cause little mortality, but it is likely that applying paint spots to mosquito wings affects behaviour and possibly survival [2]. Droplet size and visibility needs to be balanced in order to ensure that the mosquito is unaffected and able to be seen.

The effects of marking adults with powders or stains, or any other substance, should always be carefully evaluated by comparing mortalities of marked and unmarked mosquitoes of the same species, sex and if possible same age over the lifetime of the insect. Unfortunately it appears from the literature that in many experiments where marking was carried out, the effects of the marking were not evaluated statistically, or inadequately so [2]. In order to improve marking efficiency for future dispersal and population studies, methods should be rigorously compared. This study was conducted to determine the best method and colour for marking mosquitoes for MRR experiments by comparing the mortalities of marked and unmarked individuals, whether immobilising the mosquitoes had adverse effects on survival and calculating how efficient each method is in marking.

## Methods

### Mosquitoes

*Aedes aegypti* (Linnaeus) were used for all laboratory experiments. For the rearing of mosquitoes, second instar larvae were placed in plastic trays and fed daily with fish food (TetraMinBaby^©^). Pupae were transferred to a cup of water in an insect rearing cage. Larvae and adults were maintained under insectary conditions (27°C, 70% RH and a photoperiod of 12:12 [L:D] h) and provided with 10% sucrose solution.

### Marking of mosquitoes

Two or three day old *Aedes aegypti* mosquitoes were randomly aspirated into 90 mm plastic containers (1 L tumblers) with gauze tops, until there were 15–18 females and 15–18 males in each container. After applying the marking technique, all experimental containers had cotton wool soaked in 10% sucrose solution placed on top of the gauze, which was refreshed daily. A plastic lid was placed over the top of the container to keep the humidity high. Dead mosquitoes were recorded daily and removed.

Mosquitoes were immobilised prior to the marking methods to allow a better coverage, and to prevent accidental release. To check that survival was not affected by immobilising them, an experiment was set up to compare briefly immobilised mosquitoes to control mosquitoes. Two containers were placed in a freezer (-18°C) for one minute, and then changed over to a container held at room temperature (22°C) until the mosquitoes had recovered. Two containers of immobilised mosquitoes and two containers of control mosquitoes were then placed back in the insect rearing room (27°C) until all mosquitoes died.

Dusts from two companies were used in this experiment; A-19 Horizon Blue and A-13-N Rocket Red ‘DayGlo’ series A fluorescent pigments (DayGlo Color Corp, Cleveland, OH, USA), which will be referred to as ‘D Blue’ and ‘D Red’, gold metallic dust (Brian Clegg, UK), pink, blue and red fluorescent dusts (Brian Clegg, UK), which will be referred to as ‘BC Gold’, ‘BC Pink’, ‘BC Blue’ and ‘BC Red’. Yellow, blue and red fluorescent paints (Brian Clegg, UK) were also used in this experiment. DayGlo dusts are manufactured from organic dyes, incorporated into a melamine formaldehyde resin and grounded into a fine powder [2]. Brian Clegg dusts are composed of calcium/magnesium carbonate, for use as powder paints.

The marking methods used in this experiment were; placing mosquitoes in a bag with dust at the bottom and gently shaking (hereafter known as ‘bag’ method), using a bulb duster to create a small puff of dust (hereafter known as ‘bulb duster’ method), using a fan to create a small dust storm within a cage (hereafter known as ‘dust storm’ method) and lightly spraying paint in small droplets (hereafter known as ‘paint’ method). The paint solution was made by mixing 2 g dust, 200 ml paint of the same colour and 200 ml distilled water. This was repeated for each paint colour. The paint control was made up of 200 ml distilled water. After the mosquitoes had been immobilised, they were transferred to a tray and the paint solution was finely sprayed, using an atomiser, three times over the mosquitoes. Mosquitoes were then transferred to a container to recover, and placed in the insect rearing room. All DayGlo and Brian Clegg dusts were used for the bulb duster, bag and dust storm method. The bulb duster was loaded with 0.3 g dust per container; mosquitoes were transferred to a tray and sprayed four times. The bag method had 0.3 g dust placed in the bottom of the bag; immobilised mosquitoes were placed in the bag and gently shaken in the bag. Mosquitoes for the dust storm method were also placed in a bag with 0.3 g dust, a fan was used to create a dust storm within the bag. After all dusting methods, immobilised mosquitoes were gently placed back in their original container. Each method had a control, where mosquitoes were handled similarly to marked mosquitoes, but dusts or paints were not added. Each method, control and colour had three repeats, each containing at least 30 mosquitoes. The survival experiment continued until all mosquitoes died.

‘Marking efficiency’ scores were given to each colour and method used. Scores were calculated by whether dust could be seen from 20 cm away, and if dust was present on their head, thorax, abdomen, legs or wings when observed using a dissection microscope. A score of ‘1’ was given if present, or ‘0’ if not present, for each category. The sum of these categories gave the overall marking efficiency score, out of a maximum of six.

### Data analysis

The survival data was analysed using the log rank test in SPSS Version 20.0 [23] across 15, 30 and 61 days. Using different time scales allowed comparison with other studies which have examined survival over short time periods and consequently may have observed different trends. Across all experimental treatments there was a clear difference between male (N = 1260), and female (N = 1235) survival (χ^2^ = 1080.80, df = 1, p < 0.0001), therefore analysis was performed separately for each sex. For comparing methods; bag, bulb duster, dust storm and paint methods were compared to unmarked control mosquitoes. For comparing colours, D Blue, D Red, BC Blue, BC Pink, BC Red and BC Gold were compared to unmarked method controls. Yellow, blue and red paints were analysed separately to the paint controls for comparison of colours.

A generalised linear model (GLM) with quasi-poisson errors was used to test marking efficiency in relation to marking method and colour. A GLM with poisson errors showed that the data was over-dispersed. Interactions were tested for, but were not present. Mosquitoes that died early (days 1–20) and late (days 21–61) within the experiment were compared separately for marking efficiency. Males and females were combined within the model because their marking efficiencies were not significantly different (df = 1, p = 0.897). All models were plotted to see how well the model fitted the data. All graphs were plotted using R version 2.15.3 [24].

## Results

The experiment ran for 61 days, at which point all mosquitoes had died. Median longevity was 12 and 28 days for males and females, respectively. Overall, 716 (58%) of females survived beyond 30 days, but only 21 (2%) male individuals survived beyond 30 days. There were no significant differences in longevity between immobilised mosquitoes and their controls for males (χ^2^ = 0.82, df = 1, p = 0.364) or females (χ^2^ = 2.21, df = 1, p = 0.137).

**Figure 1:**
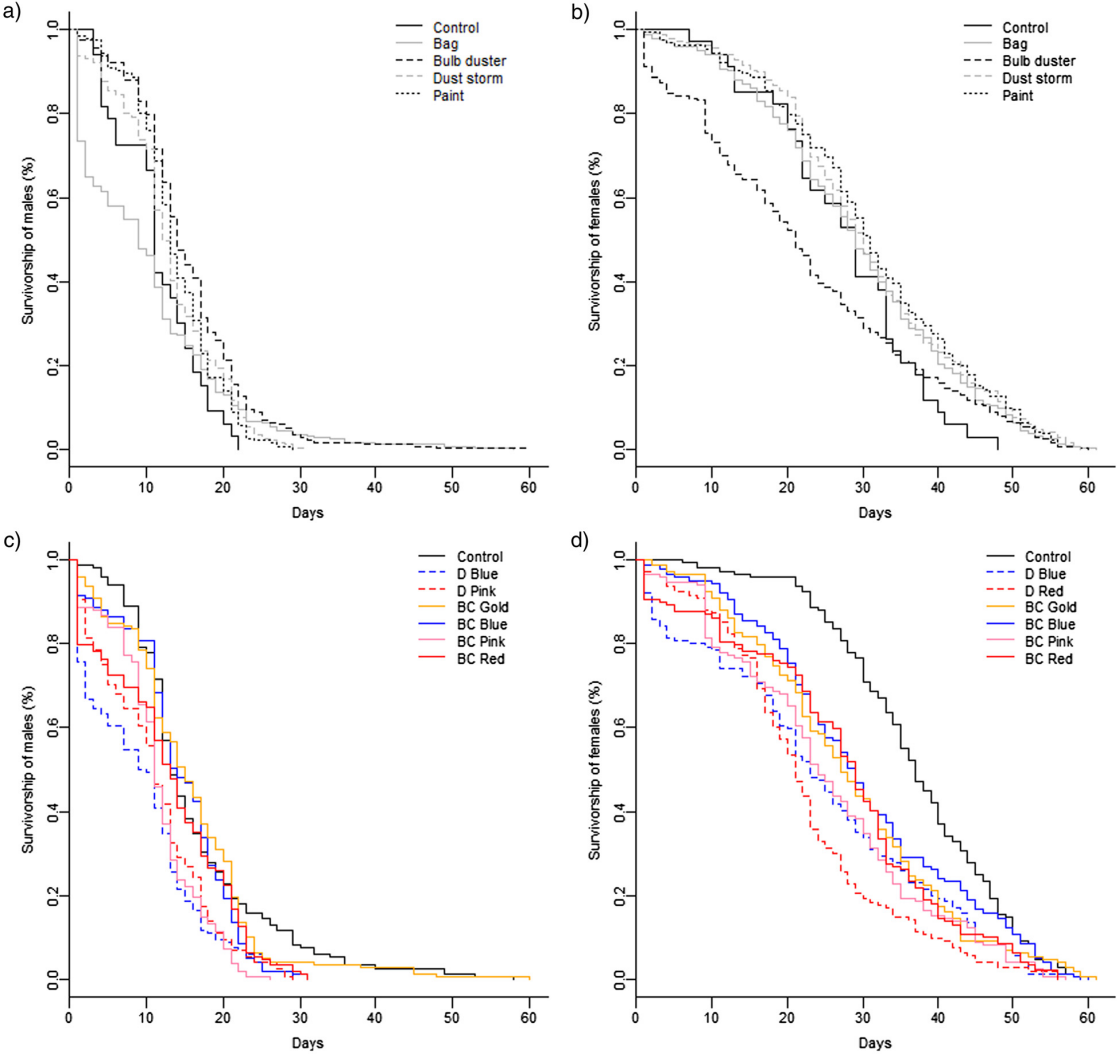
**Survivorship plots of gender, colour and method.** Survivorship plot of **a)** male and **b)** female *Aedes aegypti* marked using different methods (bag, bulb duster, dust storm & paint) vs. unmarked controls. Survivorship plot of **c)** male and **d)** female mosquitoes marked with DayGlo colour dust (D Blue & D Red) and Brian Clegg coloured dust (BC Blue, BC Pink, BC Red, BC Gold) vs. control mosquitoes.

**Figure 2:**
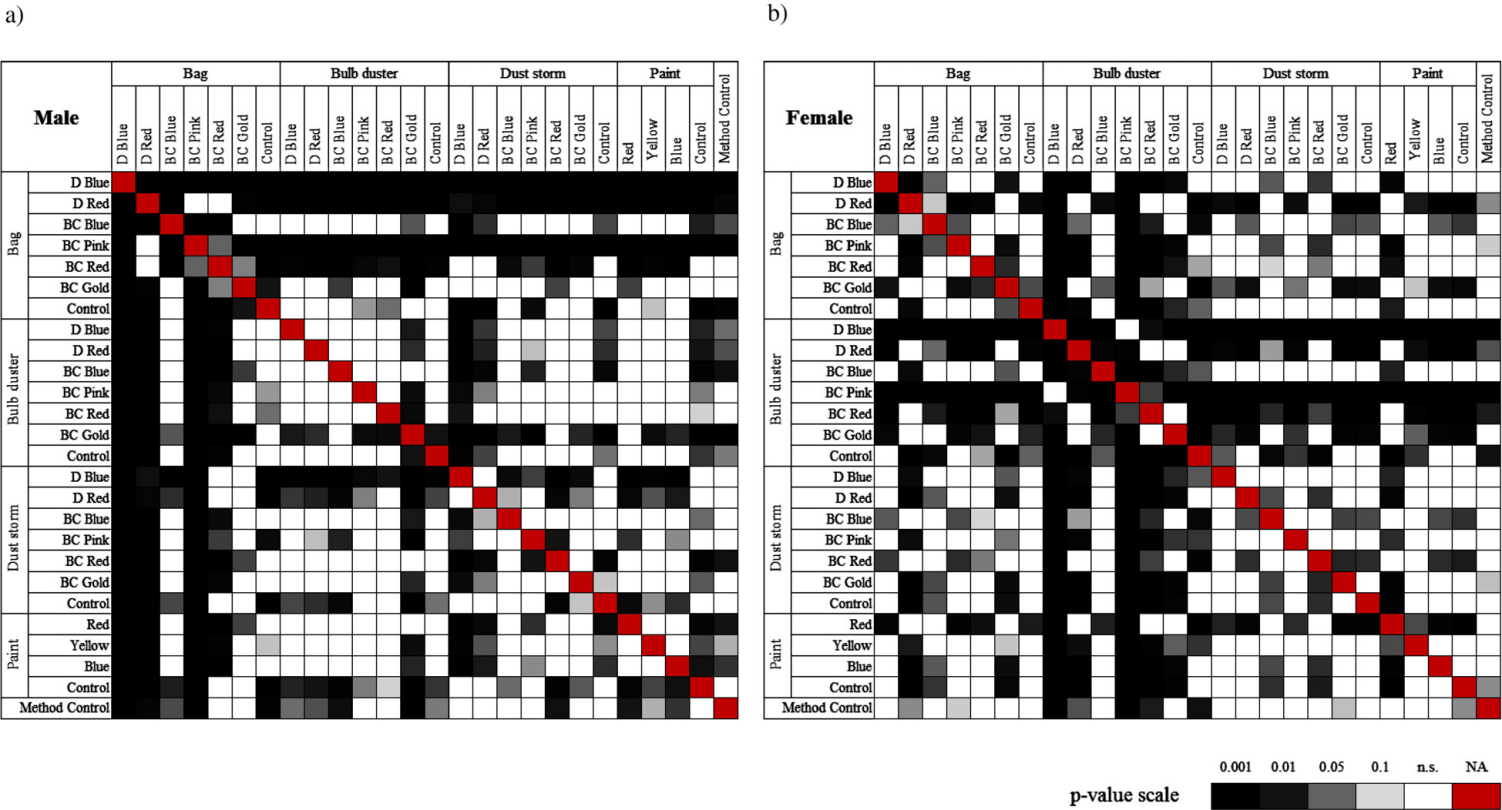
**Pairwise treatment comparison of significance.** Surface plot showing pairwise patterns of significance between different treatments for the first 15 days of **a)** male and **b)** female survival.

The bag method for males showed a dramatic decline in survivorship during the first 15 days of the experiment (Figure 1a), and was the only method that was significantly different to the control (Table 1). Bulb duster, dust storm and paint methods had significantly higher longevity in males than the control, but the bag did not (Table 1). Contrary to males, females showed a dramatic decline in survivorship in the first 15 days of the experiment for the bulb duster method (Figure 1b), which was the only method significantly different to the control (Table 1). Pairwise comparisons of treatments showed that marking method was the dominant determinant of survival in comparison to other treatments (Figure 2).

**Table 1:** **Log-rank tests for *Aedes aegypti *** **marked using dusts, paints and different marking methods**

			**Males: 15 days**		**Males: 30 days**		**Females: 15 days**		**Females: 30 days**	
	**N**	**df**	**χ**^**2**^	**p-value**	**χ**^**2**^	**p-value**	**χ**^**2**^	**p-value**	**χ**^**2**^	**p-value**
**Method**										
Bag	326	4	5.77	0.016*	0.31	0.577	1.21	0.271	2.04	0.153
Bulb duster	335		1.66	0.197	10.65	0.001***	5.17	0.023*	6.35	0.012*
Dust storm	338		0.02	0.886	3.11	0.078 .	0.42	0.518	0.16	0.690
Paint	195		1.19	0.275	5.03	0.025*	1.24	0.265	0.19	0.661
**Colour (dust)**										
D Blue	144	6	16.493	0.000***	15.70	0.000***	3.732	0.053 .	23.12	0.000***
D Red	144		14.689	0.000 ***	8.72	0.003***	0.528	0.467	24.47	0.000***
BC Blue	139		4.42	0.039*	0.05	0.830	0.286	0.593	6.89	0.009***
BC Pink	148		9.824	0.002**	14.84	0.000***	0.025	0.876	16.03	0.000***
BC Red	142		6.403	0.011*	0.49	0.484	2.121	0.145	7.093	0.008***
BC Gold	138		3.343	0.068 .	0.03	0.857	0.385	0.535	16.563	0.000***
**Colour (paint)**										
BC Blue	47	3	0.37	0.543	9.843	0.002***	0.649	0.421	0.764	0.382
BC Red	51		0.393	0.531	2.793	0.095 .	4.503	0.034	0.244	0.622
BC Yellow	48		1.942	0.163	5.575	0.018*	2.882	0.900	1.073	0.300
**Company**										
DayGlo	288	2	18.609	0.000***	14.74	0.000***	1.994	0.158	27.23	0.000***
Brian Clegg	281		6.77	0.009***	0.292	0.589	1.391	0.238	8.25	0.004***

***indicates p<0.001, **p<0.01, *p<0.05, . p<0.1.

With the exception of BC Gold, the survival and longevity of mosquitoes was significantly lower for all colours compared to the control in males (Figure 1c). In females, the survival of mosquitoes was significantly lower for all colours (Figure 1d). The same trend was observed for the 15 and 30 periods in males (Table 1). For females, the first 15 days did not show a significantly different survivorship to the control, but did for the 30 day periods (Table 1). DayGlo dusts showed a higher mortality rate than the Brian Clegg dusts, but both were significantly different to the control for males and females (Table 1). For the paint colours, significant differences were not observed in the first 15 days for males and the first 15 and 30 days for females (Table 1). Blue paint significantly decreased the longevity of female mosquitoes over 61 days, but red and yellow paint increased the longevity (Table 1). Red, blue and yellow paint increased the survivorship of males for 30 days (Table 1). The 61 and 30 day periods showed similar trends for all treatments for both males and females.

**Figure 3:**
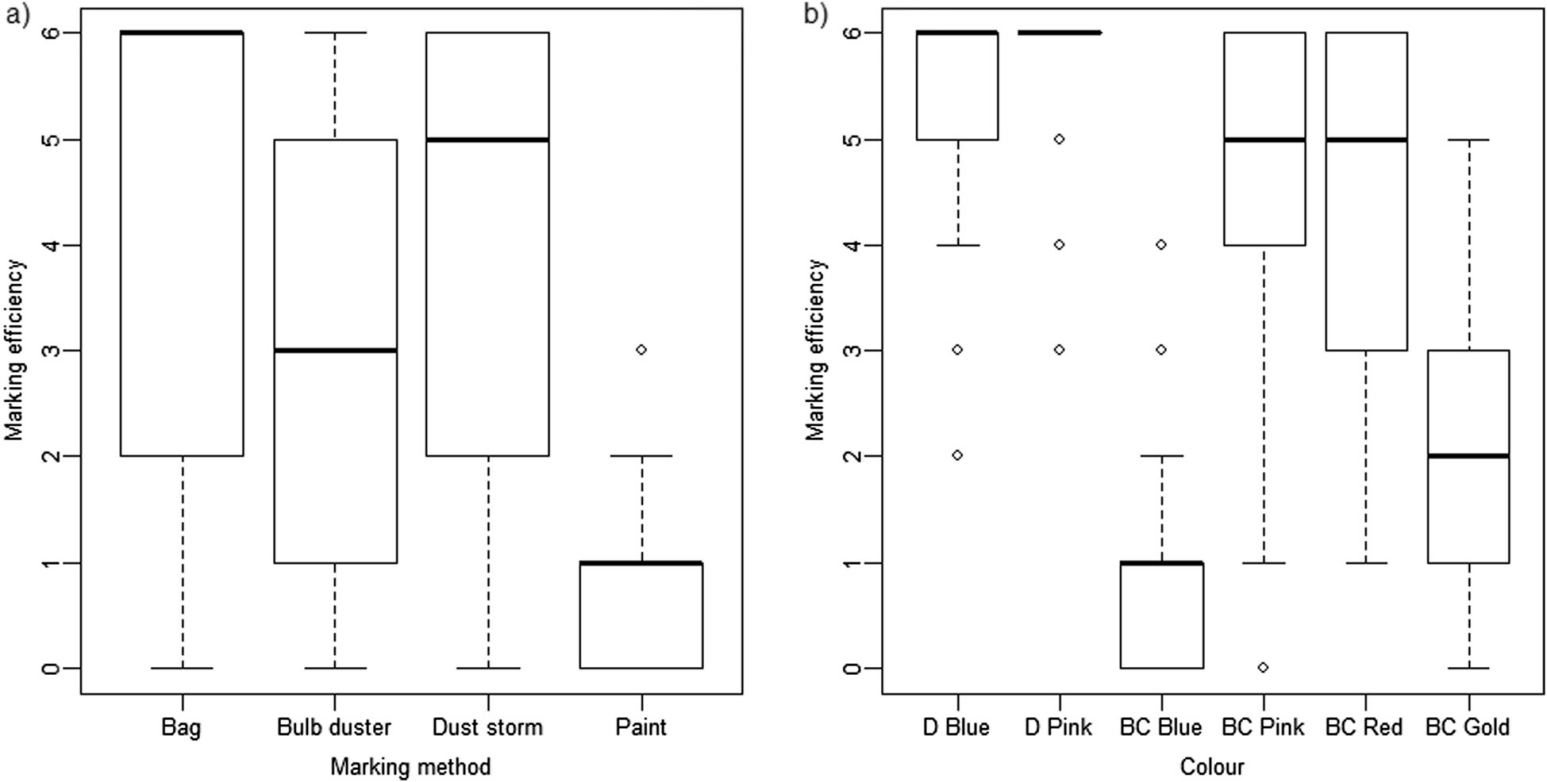
**Marking efficiency of methods and colours.** Marking efficiency (0–6) of male and female *Aedes aegypti* using different **b)** marking methods and **a)** colours of dust.

For marking efficiency, no statistical difference was observed between early (days 1–20) and late (days 21–61) survival, indicating that those individuals who showed signs of being marked in the beginning of the experiment remained so for the rest of the experiment (df = 1, p < 0.0001). All methods of application and colours were visible (p ≤ 0.002 for all dusting combinations), but some more visible than others (Figure 3). The paint method was the least visible, followed by the bulb duster (Figure 3a). D Blue, D Red, BC Red and BC Pink showed the greatest marking efficiency (Figure 3b). DayGlo dusts had a higher marking efficiency than Brian Clegg dusts.

## Discussion

The effect of dusts on insect survival and behaviour varies with species [25] thus the amount of dust used and application technique is likely to have an effect. Although some studies have found no significant differences between the survival rates of marked insects to unmarked controls [25-39], others have documented adverse dusting effects [40-47], with factors including brand and colour often associated with dramatic differences between marked and unmarked controls.

This study found that males and female *A. aegypti* were adversely affected by the bag and the bulb duster methods. It is possible that the bag method was more damaging to males due to their fragility and that physical shaking damaged their extremities. Shaking procedures have shown to be detrimental to delicate insects because they place too much dust on the insects, and cause high mortality immediately after marking [1,48]. It is less certain as to why the bulb duster method gave greater mortality for females but it may also be due to an excess of dust that female mosquitoes were unable to groom off. Too much dust can decrease mobility, interfere with sensory organs and increase mortality [49]. Excessive moisture whilst marking can cause insects to become ‘gummed up’ with dust [26], but this was not an issue in this study as relative humidity was constant and there were no observations of any water droplet formation or gumming of dust.

The commonly used dust storm method had less impact on survival and thus is better than the other two dusting methods. This is perhaps due to dust storms atomising the dust better than the bag and bulb dusting methods. As for marking efficiency for each method, the dust storm and bag provided the greatest coverage of mosquitoes. Spraying mosquitoes with fluorescent paint had no marked effect on survival, possibly due to the small droplet use in the study, which was designed to balance survival and coverage. The paint method showed a low marking efficiency in comparison to the dry marking methods. This could be increased by extra sprays during marking, but too much moisture can affect behaviour and survival [2]. Whilst relative humidity prolongs mosquito survival, excess wetting mosquitoes can be a source of mortality [26], so this method must be used with caution.

Colour choice appears to be important for MRR studies. The reds and pinks used had intermediate values for both survival and marking efficiency. Although D Blue could be seen using the naked eye, the significant negative effect on survival makes it unsuitable for studies where an assumption is made that the marked individuals are of equal fitness to the unmarked. Other shades of blue which have a different chemical make-up and/or different concentrations of agents may not create this effect. This was observed in *Reticulitermes* which had a significant mortality rate over 15 days for Sudan Red 7B but not Neutral Red [50]. This may contribute to the success and failure of any MRR studies which use different shades of D Blue or other colours, although many other factors should be considered such as the scale, location, season, duration of the study and marking method. The low impact of BC Blue on survival could be used as evidence to argue this but owing to its poor marking efficiency, it is possible that the dust was unable to adhere to individuals and was thereby spuriously associated with high survivorship. A colour change occurred in BC Gold tests with blue webbing across appendages at 15 days and it additionally had greater longevity for those marked with this colour for reasons unknown.

Mosquitoes marked with the two particular shades of DayGlo dust had reduced survival, but further testing with more colours, shades and chemical compositions is required to conclude whether one brand has lower mortality. Other studies have also observed reduced survival rates that are dependent on the colour of dust used. Coviella *et al.*[45] observed reduced survival in cicadellids, but only for one of the six colours of DayGlo dusts tested. One of 14 colours of powders tested by Toepfer *et al.*[51] reduced the survival in corn rootworm adults.

Two concerns in scaling the results from this laboratory study up to a semi-field or field trial are establishing a practical immobilisation method and preventing contact-transfer among marked and unmarked individuals. Firstly, using a freezer to immobilise mosquitoes in the field would prove difficult in certain situations, but diethyl ether is a suitable replacement. Even though freezing was chosen here over a chemical agent, provided the knock-out time is similar and the chemical has no lasting effects, it is likely to have no significant effect in the survival of the mosquito [2]. As it is well established that many species and strains vary in environmental resilience and excessive immobilising methods have the potential to cause significant mortality, immobilisation and marking technique should be tested in the laboratory and suitable concentrations established before field experiments. It should also be noted that marking under a laboratory setting without the uncontrolled weather and environmental conditions of a field study are likely to mean that marking efficiency will be high for a longer duration than can be expected in the field.

Secondly, contact-transfer can bias results when using methods which heavily rely on dust coverage, potentially marking 1-3% of the unmarked mosquitoes after 24 hours if exposed to DayGlo mosquitoes in a confined space [20]. Dust particles can potentially be transferred to unmarked insects in traps and sweep nets used for sampling [52], where individuals are forced to come into contact with each other, but is not an issue with sticky traps. It should be noted that same-sex transfer is likely to be of little concern and most transfer would occur during mating. Crumpacker *et al.*[26] observed no dust transfer during mating of *Drosophila pseudoobscura* or following the crowding of heavily marked and unmarked individuals after they were allowed to clean themselves before mixing. The implication of this for field studies is that they should mark individuals then give them time to clean themselves in relatively uncrowded conditions before releasing them.

We expect it is likely that the effects we observed here of dusting methods on survival of *Ae. aegypti* will be similar to the effects on *Anopheles* species, although other experiments have shown little difference in survival of marked and unmarked *An. punctulatus, An. maculatus, An. sinensis, An. subpictus* and *An. stephensi*[6,31,39,52-54]. The survival of a mosquito is dependant on many variables, including its size and resistance to environmental stress [55-57]. The effects of marking represents an additional stress on the body of the insect, making it necessary to carry out this experiment on other medically-important mosquito subfamilies, including the malaria vector species in *Anopheles*, where a range of body sizes and survival rates have been reported [2,58,59].

We found that application method had a significant effect on mosquito survival, and that the dust storm method caused the least mortality. Of the colours, the two shades of blue we tested should be avoided. For increased survival and marking efficiency, BC Pink or BC Red appear to be the most viable options. Care should be taken before using new colours (e.g. BC Gold) and when assuming there are no significant effects on mosquito survival with a chosen method, as our results suggest they may strongly bias the results of a MRR study. Choosing the best technique for use in the field will be essential to the success of field-based studies in mosquito dispersal.

## Conclusions

Few studies have addressed the implications of marking efficiency and survivorship on male and female mosquitoes following marking, and even fewer have compared marking methods. *Aedes aegypti* is the primary vector of dengue, and its spatial movement is of interest to many policymakers and modellers. With no specific treatment, and the increased global incidence of dengue, it is becoming paramount to understand the vector’s dispersal through studies such as MRR. Successful MRR studies require a benign technique that adheres to the mosquito for a defined duration. This study showed that treatments not only affected males and females differently across 15, 30 and 61 days, but also particular colours and methods were significantly different to controls. Males dusted with D Blue and females with D Red had the most significant reduction in survivorship in comparison to the control. Dusting using BC Red or BC Pink showed both reasonable performance in marking and impact on overall survival across males and females. Overall, the dust storm method provided the best trade-off between survival and marking efficiency.

## Abbreviations

MRR: Mark-release-recapture study; D (colour): DayGlo colour; BC (colour): Brian clegg colour.

## Competing interests

The authors declare that they have no competing interests.

## Authors’ contributions

Both authors participated in the design of the study, performed the statistical analyses, read and approved the final manuscript.

## References

[B1] Hagler JR Jackson CG Methods for marking insects: current techniques and future prospects Annu Rev Entomol 2001 46 511 543 10.1146/annurev.ento.46.1.511 11112178

[B2] Silver JB Mosquito Ecology: Field Sampling Methods 2008 3 London: Springer

[B3] Southwood TRE Henderson PA Ecological Methods 2000 3 Oxford: Blackwell Science

[B4] Darling ST Entomological research in malaria South Med J 1925 18 446 449 10.1097/00007611-192506000-00017

[B5] Sheppard PM MacDonald WW Tonn RJ Grab B The dynamics of an adult population of *Aedes aegypti* in relation to dengue haemorrhagic fever in Bangkok J Anim Ecol 1969 38 661 702 10.2307/3042

[B6] Reisen WK Mahmood F Parveen T *Anopheles subpictus* Grassi: observations on survivorship and population size using mark-release-recapture and dissection methods Res Popul Ecol 1979 21 12 29 10.1007/BF02512636

[B7] Muir LE Kay BH *Aedes aegypti* survival and dispersal estimated by mark-release-recapture in northern Australia Am J Trop Med Hyg 1998 58 277 282 9546403 10.4269/ajtmh.1998.58.277

[B8] Russell RC Webb CE Williams CR Richie SA Mark-release-recapture study to measure dispersal of the mosquito *Aedes aegypti* in Cairns, Queensland, Australia Med Vet Entomol 2005 19 451 457 10.1111/j.1365-2915.2005.00589.x 16336310

[B9] Williams CR Bader CA Williams SR Whelan PI Adult mosquito trap sensitivity for detecting exotic mosquito incursions and eradication: a study using EVS traps and the Australian southern saltmarsh mosquito, *Aedes camptorhynchus* J Vector Ecol 2012 37 110 116 10.1111/j.1948-7134.2012.00207.x 22548544

[B10] Welch CH Kline DL Allan SA Barnard DR Laboratory evaluation of a dyed food marking technique for *Culex quinquefasciatus* (Diptera: Culicidae) J Am Mosq Control Assoc 2006 22 626 628 10.2987/8756-971X(2006)22[626:LEOADF]2.0.CO;2 17304928

[B11] Tsuda Y Komagata O Kasai S Hayashi T Nihei N Saito K Mizutani M Kunida M Yoshida M Kobayashi M A mark-release-recapture study on dispersal and flight distance of *Culex pipiens pallens* in an urban area of Japan J Am Mosq Control Assoc 2008 24 339 343 10.2987/5754.1 18939684

[B12] Conway GR Trpis M McClelland GAH Population parameters of the mosquito *Aedes aegypti* (L.) estimated by mark-release-recapture in a suburban habitat in Tanzania J Anim Ecol 1974 43 289 304 10.2307/3366

[B13] Cho S-H Lee H-W Shin E-H Lee H-I Lee W-G Kim C-H Kim J-T Lee J-S Lee W-J Jung G-G Kim T-S A mark-release-recapture experiment with *Anopheles sinensis* in the northern part of Gyeonggi-do, Korea Korean J Parasitol 2002 40 139 148 10.3347/kjp.2002.40.3.139 12325443 PMC2721040

[B14] Wilkins EE Smith SC Roberts JM Benedict M Rubidium marking of *Anopheles* mosquitoes detectable by field-capable X-ray spectrometry Med Vet Entomol 2007 21 196 203 10.1111/j.1365-2915.2007.00683.x 17550439

[B15] Lindquist AW Ikeshoji T Grab B De Meillon B Khan ZH Dispersion studies of *Culex pipiens fatigans* tagged with ^32^P in the Kemmendine area of Rangoon, Burma Bull World Health Organ 1967 36 21 37 4227195 PMC2476345

[B16] Hamer GL Donovan DJ Hood-Nowotny R Kaufman MG Goldberg TL Walker ED Evaluation of a stable isotope method to mark naturally-breeding larval mosquitoes for adult dispersal studies J Med Entomol 2012 49 61 70 10.1603/ME11076 22308772 PMC4106289

[B17] Jones CE Lounibos LP Marra PP Kilpatrick AM Rainfall influences survival of *Culex pipiens* (Diptera: Culicidae) in a residential neighbourhood in the mid-Atlantic USA J Med Entomol 2012 49 467 473 10.1603/ME11191 22679852 PMC3375620

[B18] Ikeshoji T Yap HH Impact of the insecticide-treated sound traps on an *Aedes albopictus* population Jap J Sanit Zool 1990 41 213 217

[B19] Renshaw M Service MW Birley MH Host finding, feeding patterns and evidence for a memorized home range of the mosquito *Aedes cantans* Med Vet Entomol 1994 8 187 193 10.1111/j.1365-2915.1994.tb00162.x 8025329

[B20] Fryer JC Meek CL Further studies on marking an adult mosquito, *Psorophora columbiae*, in situ, using fluorescent pigments Southwest Entomol 1989 14 409 418

[B21] Myles TG Grace JK Behavioural ecology of the eastern subterranean termite in Ontario as a basis for control Proc Technol Transfer Conf 1991 2 16 27

[B22] Forschler BT Fluorescent spray paint as a topical marker on subterranean termites (Isoptera: Rhinotermitidae) Sociobiology 1994 24 27 38

[B23] Corp IBM IBM SPSS Statistics for Windows. Version 20.0 2011 New York: Armonk

[B24] R Development Core Team R: A language and Environment for Statistical Computing [http://www.R-project.org]

[B25] Narisu Lockwood JA Schell SP A novel mark-recapture technique and its application to monitoring the direction and distance of local movements of rangeland grasshoppers (Orthoptera: Acrididae) in the context of pest management J Appl Ecol 1999 36 604 617 10.1046/j.1365-2664.1999.00421.x

[B26] Crumpacker DW The use of micronized fluorescent dusts to mark adult *Drosophila pseudoobscura* Am Midl Nat 1974 91 118 129 10.2307/2424515

[B27] Moth JJ Barker JSF Micronized fluorescent dusts for marking *Drosophila* adults J Nat Hist 1975 9 393 396 10.1080/00222937500770291

[B28] Sempala SDK The ecology of *Aedes (Stegomyia) africanus* (Theobald) in a tropical forest in Uganda: mark-release-recapture studies on a female adult population Int J Trop Insect Sci 1981 1 211 224 10.1017/S1742758400000448

[B29] Lysyk TJ Axtell RC Estimating numbers and survival of house flies (Diptera: Muscidae) with mark/recapture methods J Econ Entomol 1986 79 1016 1022 3745629 10.1093/jee/79.4.1016

[B30] Oloumi-Sadeghi H Levine E A simple, effective, and low-cost method for mass marking adult western corn rootworms (Coleoptera: Chrysomelidae) J Entomol Sci 1990 25 170 175

[B31] Chiang GL Loong KP Chan ST Eng KL Yap HH Capture-recapture studies with *Anopheles maculatus* Theobald (Diptera: Culicidae) the vector of malaria in Peninsular Malaysia Southeast Asian J Trop Med Public Health 1991 22 643 647 1687932

[B32] Takken W Charlwood JD Billingsley PF Gort G Dispersal and survival of *Anopheles funestus* and *A. gambiae* s.l. (Diptera: Culicidae) during the rainy season in southeast Tanzania Bull Entomol Res 1998 88 561 566 10.1017/S0007485300026080

[B33] Watson TM Saul A Kay BH *Aedes notoscriptus* (Diptera: Culicidae) survival and dispersal estimated by mark-release-recapture in Brisbane, Queensland, Australia J Med Entomol 2000 37 380 384 10.1603/0022-2585(2000)037[0380:ANDCSA]2.0.CO;2 15535581

[B34] Cameron PJ Walker GP Penny GM Wigley PJ Movement of potato tuberworm (Lepidoptera: Gelechiidae) within and between crops, and some comparisons with diamondback moth (Lepidoptera: Plutellidae) Environ Entomol 2002 31 65 75 10.1603/0046-225X-31.1.65

[B35] Weldon CW Marking Queensland fruit fly, *Bactrocera tryoni* (Froggatt) (Diptera: Tephritidae) with fluorescent pigments: pupal emergence, adult mortality, and visibility and persistence of marks Gen Appl Entomol 2005 34 99 108

[B36] Nakata T Effectiveness of micronized fluorescent powder for marking citrus psyllid, *Diaphorina citri* Appl Entomol Zool 2008 43 33 36 10.1303/aez.2008.33

[B37] Hoddle MS Millar JG Hoddle CD Zou Y McElfresh JS Lesch SM Field optimization of the sex pheromone of *Stenoma catenifer* (Lepidoptera: Elachistidae): evaluation of lure types, trap height, male flight distances, and number of traps needed per avocado orchard for detection Bull Entomol Res 2011 101 145 152 10.1017/S0007485310000301 21034517

[B38] Johnson PH Spitzauer V Ritchie SA Field sampling rate of BG-Sentinel traps for Aedes aegypti (Diptera : Culicidae) in Suburban Cairns, Australia J Med Entomol 2012 49 29 34 10.1603/ME11116 22308768

[B39] Liu Q Liu X Zhou G Jiang J Guo Y Ren D Zheng C Wu H Yang S Liu J Li H Li H Li Q Yang W Chu C Dispersal range of *Anopheles sinensis* in Yongcheng City. China by mark-release-recapture methods PloS One 2012 7 e51209 10.1371/journal.pone.0051209 23226489 PMC3511368

[B40] Moffitt HR Albano DJ Codling moths: fluorescent powders as markers Environ Entomol 1972 1 750 753

[B41] LaBrecque GC Bailey DL Meifert DW Weidhaas DE Density estimates and daily mortality rate evaluations of stable fly *Stomoxys calcitrans* (Diptera: Muscidae) populations in field cages Can Entomol 1975 107 597 600 10.4039/Ent107597-6

[B42] Williams DF LaBrecque GC Patterson RS Effect of gamma rays and/or fluorescent pigments on sterility and survival of the stable fly Fla Entomol 1977 60 297 299 10.2307/3493929

[B43] Naranjo SE Influence of two mass-marking techniques on survival and flight behavior of *Diabrotica virgifera virgifera* (Coleoptera: Chrysomelidae) J Econ Entomol 1990 83 1360 1364

[B44] Dye C Davies CR Lainson R Communication among phlebotomine sandflies: a field study of domesticated *Lutzomyia longipalpis* populations in Amazonian Brazil Anim Behav 1991 42 183 192 10.1016/S0003-3472(05)80549-4

[B45] Coviella CE Garcia JF Jeske DR Redak RA Luck RF Feasibility of tracking within-field movements of *Homalodisca coagulata* (Hemiptera: Cicadellidae) and estimating its densities using fluorescent dusts in mark-release-recapture experiments J Econ Entomol 2006 99 1051 1057 10.1603/0022-0493-99.4.1051 16937655

[B46] Reid TG Reid ML Fluorescent powder marking reduces condition but not survivorship in adult mountain pine beetle Can Entomol 2008 140 582 588 10.4039/n08-035

[B47] de Guzman LI Frake AM Rinderer TE Marking small hive beetles with thoracic notching: effects on longevity, flight ability and fecunditiy Apidologie 2012 43 425 431 10.1007/s13592-011-0107-8

[B48] Meyerdirk DE Hart WG Burnside J Marking and dispersal study of adults of the citrus blackfly, *Aleurocanthus woglumi* Southwest Entomol 1979 4 325 329

[B49] Davey JT A method of marking isolated adult locusts in large numbers as an aid to the study of their seasonal migrations Bull Entomol Res 1956 46 797 802 10.1017/S0007485300037056

[B50] Su N-Y Ban PM Scheffrahn RH Evaluation of twelve dye markers for population studies of the eastern and Formosan subterranean termite (Isoptera: Rhinotermitidae) Sociobiology 1991 19 349 362

[B51] Toepfer S Levay N Kiss J Suitability of different fluorescent powders for mass-marking the Chrysomelid, *Diabrotica virgifera virgifera* LeConte J Appl Entomol 2005 129 456 464 10.1111/j.1439-0418.2005.00979.x

[B52] Miller LR Fluorescent dyes as markers in studies of foraging biology of termite colonies (Isoptera) Sociobiology 1993 23 127 134

[B53] Reisen WK Aslamkhan M A release-recapture experiment with the malaria vector, *Anopheles stephensi* Liston, with observations on dispersal, survivorship, population size, gonotrophic rhythm and mating behaviour Ann Trop Med Parasitol 1979 73 251 269 496476 10.1080/00034983.1979.11687255

[B54] Charlwood JD Graves PM Birley MH Capture-recapture studies with mosquitoes of the group of *Anopheles punctulatus* Dönitz (Diptera: Culicidae) from Papua New Guinea Bull Entomol Res 1986 76 211 227 10.1017/S000748530001470X

[B55] Haramis LD Increased adult size correlated with parity in *Aedes triseriatus* Mosquito News 1983 43 77 79

[B56] Kitthawee S Edman JD Upatham ES Relationship between female *Anopheles dirus* (Diptera: Culicidae) body size and parity in a biting population J Med Entomol 1992 29 921 926 1460629 10.1093/jmedent/29.6.921

[B57] Ameneshewa B Service MW Resting habits of *Anopheles arabiensis* in the Awash River valley of Ethiopia Ann Trop Med Parasitol 1996 90 515 521 8915128 10.1080/00034983.1996.11813077

[B58] Petrarca V Sabatinelli G Touré YT Di Deco MA Morphometric multivariate analysis of field samples of adult *Anopheles arabiensis* and *An. gambiae* s.s. (Diptera: Culicidae) J Med Entomol 1998 35 16 25 9542341 10.1093/jmedent/35.1.16

[B59] Castillo J Brown MR Strand MR Blood feeding and insulin-like peptide 3 stimulate proliferation of hemocytes in the mosquito *Aedes aegypti* PLoS Pathog 2011 7 e1002274 10.1371/journal.ppat.1002274 21998579 PMC3188524

